# Veratrum in Pneumonia

**Published:** 1872-09

**Authors:** W. H. C. Blalock

**Affiliations:** Barnesville, Georgia


### VERATRUM IN PNEUMONIA.

BY W. H. C. BLALOCK, M.D., BARNESVILLE, GEORGIA.

It is not my intention, in this article, to consume unnecessary space, or to worry the minds of your many readers. I desire "*simply*" to present my experience and views in the administration of this valuable medicinal agent in the treatment of pneumonia.

It is presumable that most of American practitioners understand that veratrum, as an arterial sedative, stands at the head of all known remedies, and is of great value in all affections attended with a forcible action of the heart. Notwithstanding this, we but seldom see anything in our journals in regard to its administration—thus leaving the young and inexperienced to grope their way in the dark.

Veratrum, since its discovery, has met with a great deal of opposition, not only from the people, but from the medical profession. The cause of the opposition in the medical profession is the ignorance of the doctor. When I hear a physician say that he does not administer veratrum in pneumonia, or any other febrile affection, I am satisfied that that man does not know how to combat disease *scientifically, rationally,* or *successfully.* The young and inexperienced doctor has a great deal of timidity in administering this potent remedy. He is aware of its importance, but he hesitates, knowing of its violent and prostrating effects when administered in an overdose; and should he venture to give it in the manner prescribed by Dr. Norwood, he will surely, nine times out of ten, produce violent prostration, and, as a consequence, he will be forced to lay it aside.

I propose to give your readers the plan I have adopted for the last four years in the administration of veratrum, and the success I have met with in pneumonia, hoping thereby to benefit the young and inexperienced physician.

Veratrum is prompt in its action, never accumulating in the system, and never failing to have the desired effect when administered judiciously, making it a valuable and indispensable remedy. Dr. Norwood's plan of administration has not proved successful in my hands. The dose as prescribed by him—six to eight drops every three or four hours—is not reliable, and has a tendency to prejudice the patient and doctor against it; for he will surely produce violent prostration if he prescribes it according to Dr. Norwood.

I will give you the history of a few cases as occurring in my practice, and the treatment.

Case I.—I was called to see, on December 3d, 1870, Miss W., aged seventeen, good constitution, etc. I found her laboring under pneumonia, with the most violent symptoms, viz: Pulse one hundred and thirty-five; temperature one hundred and five; violent pain in left side; distressing cough, with brickdust expectoration; slight delirium; physical exploration revealed marked dullness on percussion, and crepitant rale.

*Treatment.*—Gave her at once fifteen grains calomel, with one-fourth grain of morphine; applied blister to the affected side, etc.

Dec. 4.—Patient no better, the medicine having acted well during the night; pulse and temperature same as yesterday. Administered veratrum and syrup squills, half and half, in fourteen drops (making seven of veratrum), every three hours, decreasing the dose one drop at each administration, until I had reduced the dose to four drops, which I then continued without any diminution. In eight hours, I had my patient under its influence, without any unpleasant symptoms. Pulse reduced to one hundred; skin moist and pleasant; resting quietly and apparently better. I decided through the request of her parents to remain with her during the night continuing the treatment, with one-fourth grain morphine every eight hours.

Dec. 5.—Patient passed a quiet night. Pulse ninety-four; skin cool; cough not so troublesome; bloody expectoration almost gone; no pain in side, and, to use her own language, she is much better. I left her, with directions to continue the treatment until I returned.

Dec. 6.—Found my patient still improving. Pulse eighty-four, having passed a comfortable night. I continued the treatment for two days, at which time I visited her, and found her laughing and talking. I suspended treatment, and dismissed the case. She made a rapid recovery, and was walking about in ten days from the time she was taken.

Case II.—Visited Alfred H., colored, on January 20th, and found him with pneumonia. The disease had passed into the second stage. Physical exploration revealed bronchial respiration, bronchophony, and dullness on percussion. Pulse one hundred and twenty-five; rapid breathing; distressing cough; temperature one hundred and four; restlessness; slight delirium, etc.

*Treatment*—Bowels had been moved by blue mass and castor oil before my arrival. I determined to lose no time. Gave him at once seven drops of veratrum, with one-fourth grain morphine, as in the preceding case—giving the former every three hours, and the latter every night.

Visited him next morning, January 21st, and found the veratrum and morphine acting like a charm. Passed a quiet night, without any unpleasant symptoms from the veratrum. Pulse reduced to ninety-eight; skin cool; perfectly rational; cough not so distressing. I left him, with directions to continue the treatment, reducing the morphine twelve hours.

I did not visit him any more until several days, when I found him setting up, but still taking the veratrum, his morphine having given out. He had experienced no unpleasant symptoms from the medicine. I discharged him on the 25th, almost well.

Case III.—This case was one of unusual interest. Mr. B. was taken with a violent chill on September 23d. I saw him immediately, or in a few hours, and found him with well-developed pneumonia in left lung; dullness on percussion; crepitant rale; some pain in left side; distressing cough; extreme restlessness; pulse one hundred and thirty; temperature one hundred and seven.

I determined to try the abortive powers of veratrum in this case. I therefore gave him, as is always my custom with adults, seven drops veratrum every three hours, diminishing one drop as in the preciding case, with one-fourth grain morphine every eight hours; blister to the side, etc. I remained by his bedside during the day.

I had him fully under the influence of the drug in eight hours, without any nausea or vomiting. Pulse one hundred; resting quiet; severity of pain mitigated to some extent; skin more pleasant, and he is apparently better. Treatment continued during the night. Rested well; pulse ninety-four; pain entirely gone; feeling much better.

Sept. 24.—Patient still improving; treatment continued, without any inconvenience.

Sept. 25.—Mr. B. still improving; pulse eighty-two; passed a quiet night. Ordered the veratrum continued during the day and night.

Sept. 26.—Suspended treatment, and dismissed the case.

This case relapsed in a few days; yielded very rapidly to the same treatment.

I have this to say in conclusion—that since I have adopted the above manner of administering veratrum, I have in no single instancce witnessed any unpleasant effects, and have yet to see the case where it was not attended with the best results. I could multiply case after case, showing its good effects, but will not consume more of your space. Pneumonia, when in its incipiency, can be aborted by veratrum, and veratrum alone, without any unpleasant effects, when administered as above.

